# Pilot study: bone marrow stem cells as a treatment for dogs with chronic spinal cord injury

**DOI:** 10.1186/2050-490X-2-9

**Published:** 2014-12-12

**Authors:** Carlos Alberto Palmeira Sarmento, Marcio Nogueira Rodrigues, Renato Zonzini Bocabello, Andrea Maria Mess, Maria Angelica Miglino

**Affiliations:** School of Veterinary Medicine and Animal Sciences, University of São Paulo, São Paulo, Brazil; Cidade Universitaria-Butanta, Av. Prof. Orlando Marques de Paiva, 87, São Paulo, 05508270 Brazil

**Keywords:** Cell therapy, Dysfunction, Spinal cord, Disease

## Abstract

**Background:**

Chronic Spinal Cord injury is a common, severe, and medically untreatable disease. Since the functional outcomes of acute and experimental chronic spinal cord injury have been shown to improve with stem cell therapy, a case study was conducted to test if the application of stem cell also regenerates chronic SCI dysfunction. Transplantation of foetal bone marrow stem cells was applied in seven dogs with chronic spinal cord injury. Magnetic resonance images and assessments of symptoms according to the Olby scale were used to diagnose the severity of injury.

**Result:**

All dogs improved locomotor and sensory function when examined 90 days after surgery, and showed increased movement of the hind limbs, and were able to stand upright, as well as to take small steps. Tail tone was observed in seven dogs, pain reflexes and defecation return were observed in five dogs.

**Conclusion:**

The transplantation of bone marrow stem may be a promising, reliable and safe treatment for chronic spinal cord injury.

**Electronic supplementary material:**

The online version of this article (doi:10.1186/2050-490X-2-9) contains supplementary material, which is available to authorized users.

## Background

Spinal Cord injury (SCI) is a disease with devastating effect on dogs, including paresis or paralysis and/or urinary and faecal incontinence. Chronic SCI is regarded as a medically untreatable condition and there is no effective treatment [1–3]. Spinal cord lesions are commonly reported in veterinary medicine, especially in dogs, where they usually occur in association with traumas that are induced by prolapsed intervertebral discs or exogenous sources such as motor vehicle accidents [4, 5, 1]. In humans, between 3 and 5 people per every 100,000 are affected with SCI [6]. Clinical signs in dogs are similar to those encountered in human patients, and dogs that suffer from severe SCI have the same poor prognosis for neurological recovery as their human counterparts [7]. The severity of the neurological signs are graded as follows: grade I (only spinal hyperaesthesia), grade II (ambulatory paraperesis, ataxia, proprioceptive deficits), grade III (non-ambulatory paraparesis), grade IV (paraplegia with nociception), and grade V (paraplegia with loss of nociception) [8]. Likewise, a scale of 14 points is used to assess the severity of the disease [9].

Diagnostic imaging has become increasingly important for assessing the prognosis and determining treatment decisions with intervertebral disc extrusion [8, 10–12]. For dogs, the temporal aspects of the onset and duration of clinical signs after intervertebral disc herniation have been used as prognostic indicators [1], suggesting that clinical assessment is essential after SCI [12].

Treatments with stem cells have been performed in animal and human SCI models, with promising results [3, 13–16]. In particular, transplantation of stem cells has been shown to improve the functional outcome of experimental SCI in rats [17–19]. Pre-clinical reports indicate that the transplantation of bone marrow mesenchymal stromal cells is encouraging in acute, SCI-simulating models, including monkeys [20], humans [21] and dogs [22, 23]. The aim of the current pilot project is to evaluate if transplants of foetal canine bone marrow derived stem cells into spinal cord lesions improved the regeneration and spinal cord function in dogs with severe, chronic spinal cord lesions.

## Results

On the day following surgery, the animals did not present with any adverse symptoms. There was no formation of local oedema, seroma, or other complications. No feeding issues or associated loss of weight was noted during the investigative period. Sutures were removed after 10 days without problems.

**Figure 1:**
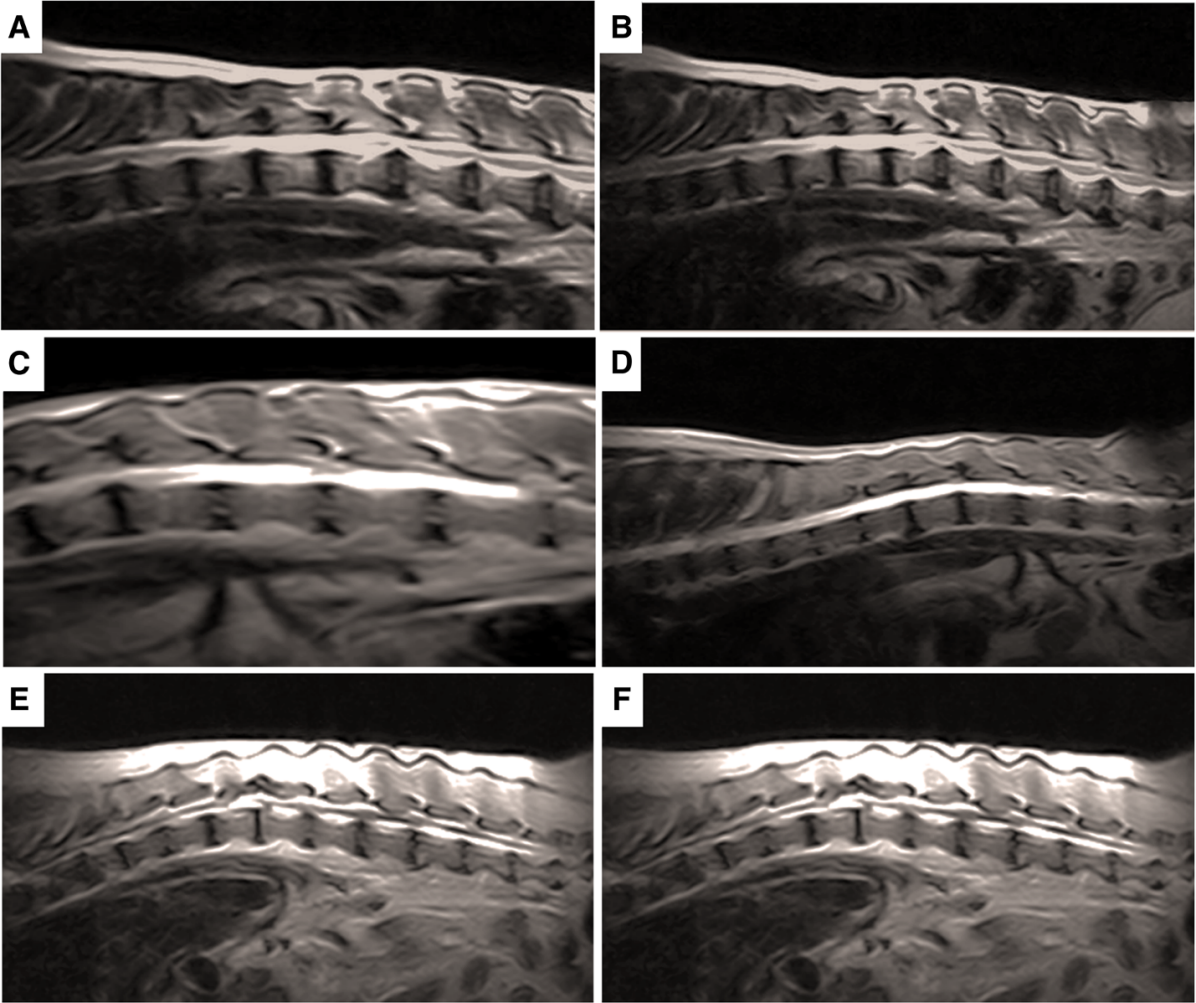
**MRI of dogs thoracolumbar segment.** In **(A)** the pre treatment the animals showed severe extradural compressive myelopathy ventrolateral between T11-T12 and disc protrusion at L3-L4, disc degeneration and spondylosis multifocal in thoracic and lumbar spine. In **(B)** after treatment the animal showed the same diagnosis at MRI without regression or progression of the previous reported. In **(C)** homogeneous hyperintensity maintained between T9-L2, extradural compressive myelopathy, mild to moderate between T12-T13, previous surgical procedure between T11-T13, with secondary muscle atrophy, disc dehydration multifocal in thoracolumbar spine were observed. In **(D)** after the treatment the same alterations were observed in the MRI. In **(E)** myelopathy extradural compressive between T9-T120 and T13-L1, with the presence of secondary siringohidromielia. In **(F)** after treatment probable thoracolumbar muscular atrophy with presence of siringohidromielia between T12-L1 and L3, and severe at T13-L1, multifocal disc dehydration in thoracolumbar spine.

**Figure 2:**
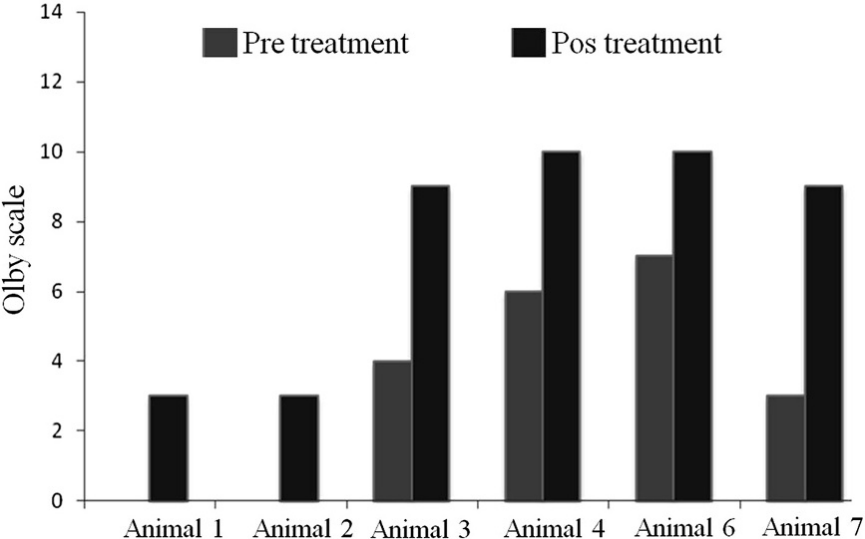
**Evolution of the animals according to the scale of Olby** [9] **.**

MRI did not show major differences after the treatment (Figure 1). All animals that were considered for statistical analysis showed significant improvement of the Olby scales after stem cell transplantation (Table 1, Figure 2). They also showed increased movement of the hind limbs on both treadmills. Animals 3, 4, 6 and 7 were able to support their own weight and to make small, uncoordinated steps and returned tail tone, superficial and deep pain reflexes as well as defaecation (Table 1). In detail, Animal 1 suffered from a flaccid paralysis without significant joint contracture that became slightly better after surgery, including movements of the hind limbs and improved muscle contraction, indicating a reactivation of the nervous pathways. Animal 2 showed sharp muscle spasm that compromised the joints of the hind limbs. After surgery, the muscles recovered, in addition to an increased range of motion (Table 1). Animal 3 had little muscle contracture before therapy, but showed improved movement of the hind limbs in the aquatic treadmill afterwards. The animal was able to hold its body weight and to protrude the tail voluntarily (Table 1). Animal 4 was the only one that was affected by trauma. Before treatment it suffered from urinary incontinence and could not stand its body. After treatment, it was able to take several steps and spontaneous voiding returned. Animal 5 had a bilateral patella dislocation, in addition to the spinal lesion, resulting in high muscle contraction that became much better after surgery. However, one knee was not stable and required further surgery during the investigated period, and a final assessment of the Olby score was not possible and the animal was not included within the statistical analysis. The animal 6 showed aggressive behavior and the postoperative evaluation of superficial and deep pain sensation and panniculus reflex were performed with the aid of a forceps. In this case and for Animal 7, a significant improvement in walking and the return of voluntary movement of the tail was observed (Table 1). Additional file 1: Videos S1 and S2 were provided to show the evolution of the one animal treated after 30, 60 and 90 days after treatment.

**Table 1:** **Group of animals showing height of the lesion by MRI and data relating to assessments of physical therapy animals, according to Olby scale before and after the surgical procedure and injection of stem cells**

Animal	Age-years	Breed	Diagnosis	Lesion	Moment		Olby scale
1	6	Teckel	Intervertebral disc extrusion	T13-L1	Pre – treatment	0	Absence of movement of the hind limbs without feeling deep pain.
					Pos – treatment	3	Minimum protraction (movement of a joint) without dislocation of the pelvic limb weight.
2	8	Teckel	Intervertebral disc extrusion	T13-L3	Pre – treatment	0	Absence of movement of the hind limbs without feeling deep pain.
					Pos – treatment	3	Minimum protraction (movement of a joint) without dislocation of the pelvic limb weight.
3	6	Lhasa Apso	Intervertebral disc extrusion	T11-12	Pre – treatment	4	Protraction of the hind limbs without displacement in a joint weight of at least 50% of the time.
					Pos – treatment	9	Protraction of the hindlimbs with weight shift in 100% of the time with reduced strength of hindlimbs. More than 90% error when walking (eg: Crossing pelvic limbs, shuffling walk, stay on station with the backs of the feet, tripping).
4	2	Mongrel	Trauma	T10-11	Pre – treatment	6	Protação hind limb with a displacement of less than 10 weight% of the time.
					Pos – treatment	10	Protraction of the hindlimbs with weight shift in 100% of the time with reduced strength of hindlimbs.
6	8	Poodle	Intervertebral disc extrusion	L1-L4	Pre – treatment	7	Protaction hind limb with a displacement of from 10 to 50 weight% of the time.
					Pos – treatment	10	Protaction of hindlimbs with weight shift in 100% of the time with reduced strength of hindlimbs.
7	10	Teckel	Intervertebral disc extrusion	T11-12	Pre – treatment	3	Minimum protraction (movement of a joint) without dislocation of the pelvic limb weight.
					Pos – treatment	9	Protraction of the hindlimbs with weight shift in 100% of the time with reduced strength of hindlimbs mm. More than 90% error when walking (eg: Crossing pelvic limbs, walking crossing feet remain on station with the backs of the feet, tripping).

## Discussion

We showed in the current case study that adult dogs with chronic SCI could be successfully treated with the transplantation of foetal canine bone marrow stem cells. Examined 90 days after surgery, all animals had increased locomotor and sensory functions according to the Olby scale, without health complications or decline in the neurologic status. The patients received stem cell treatment, but also underwent physical therapy, so it is hard to determine the exact cause of any improvements. It should be given thought, however, that many patients with chronic SCIs do not recover strength so many time post injury with physical therapy alone [24]. The data suggested that transplanted stem cells may lead to a functional recovery of SCI, similar to what was reported for acute lesions in dogs [22]. Also, it was reported in dogs, indicated by higher values of the Texas SCI scale after the transplantation of bone marrow stem cells [23]. In others studies recovery of motor and sensory functions 53 days after initial cell transplantation and the ability to stand after 79 days was reported [25]. The observed improvements in dogs are similar to reports on humans with acute SCI [21]. In addition to the applications for SCI regeneration, bone marrow stem cells have broad therapeutic significance. They have been used as donor cells for haematologic diseases in animal models [26], or to treat cardiovascular diseases and cancer in humans [27]. Also, transplanted bone marrow stem cells improve neurologic disorders in models of central nervous system injury by generating neural cells or myelin-producing cells [28]. Donor cells from male humans have been shown to rebuild neurons in the brains of female recipients [29]. In general, bone marrow stem cells differentiate into a variety of non-hematopoietic cell lineages, e.g. muscle, skin, liver, lung, cardiac myocytes, endothelial and neuronal cells [30, 31]. For stem cell therapy, the use of bone marrow-derived stem cells cause minor problems in regard to immunological rejections, graft-versus-host reactions or carcinogenesis compared to other embryonic or adult stem cells [32, 33].

## Conclusion

In addition to the former therapies, our case study indicates that foetal bone marrow stem cells represent a promising treatment for chronic SCI in canine patients, however others stem cell sources can also be considered to use for treatment, suggesting that clinical trials with more animals are needed.

## Methods

### Selection of animals and preoperative procedures

Seven dogs with chronic SCI, from different private veterinary clinics in São Paulo, were used (Table 1). The selected animals presented with paralysis, a lack of conscious proprioception, the absence of deep pain perception with presence of the withdrawal reflex in the hind limbs, assessed according to the Olby scale (see below), and diagnosis of spinal cord compression of thoracolumbar intervertebral discs between segments T10-L3 after magnetic resonance imaging (MRI). The absence of deep pain perception for more than 60 days was used as a diagnostic tool for chronic injury. The animals did not show any positive response to former treatments such as medication, physiotherapy or acupuncture. Conventional examinations (blood count, biochemistry, urinalysis, stool analysis and electrocardiogram) were performed, indicating normal values. MRI (Vet-MR Grande-Esaot) was performed to determine the precise location of the lesion. The owners signed an informed consent, making them aware of all procedures and risks associated with the surgery and cell transplantation. The project was approved by the Ethical committee of the University of Sao Paulo (Nr. 2063/2010).

### Surgery and cell transplantation

Prior to anesthesia, the animals were injected with 0.05 mg/kg acepram (acepromazine®^1^) associated with 4 mg/kg dolosal (Meperidine®^2^), both intramuscularly. After 15 minutes anaesthesia was induced with 5 mg/kg propofol (Propovan®^3^ - Cristália) intravenously and maintained by inhalational anaesthesia with isoflurane (Isoforine®^4^ - Cristália). During surgery the animals underwent dorsolateral hemilaminectomy in the injured spinal cord. The incision was made lateral to the midline and dorsal to the lesion. After incision of the skin and muscles, the fascia was incised lumbodorsal alongside the corresponding spinous processes of the vertebrae, and, with the aid of a periosteum elevator, the spinal processes were isolated. By exposing the dorsal surface of the articular process, a window was opened to provide access to the spinal canal. After which, the medullary mass was compressed and damaged tissue was removed. After durotomia, intramedullary injection of 1×10^6^ of stem cells derived from foetal canine bone marrow [31] was completed cranial to the injury, at the focal point of the lesion, and caudal to the lesion. The postoperative therapy consisted of 30 mg/kg Kefazol ® (cefazolin sodium) 30 mg/kg, 0.1 mg/kg meloxicam (Generic Biosintética) and 2 mg/kg Tramal ® (tramadol hydrochloride) for seven days.

### Postoperative examinations

Seven days after surgery, the animals were trained on aquatic and dry treadmills. To observe possible improvement, each animal was filmed in different positions 30 and 60 days after surgery (data not shown) as well as after 90 days. The dogs were scored using the Olby scale of 14 points by three veterinary physiotherapists not involved in the experiments. Dogs were considered to be able to support their own weight when they maintained their body for at least four footsteps. The reflexes of superficial and deep pain were evaluated with the help of tweezers. Statistical analyses were performed using the PRISM 4.0 statistical software package (GraphPad, Inc., San Diego, CA). Significance for all analyses was set at P-values of 0.05.

## Electronic supplementary material

Additional file 1: Videos S1 and S2: Progressive response of the animal during the treatment between the analysis performed. (ZIP 25 MB)
